# Efficient and effective assessment of deficits and their neural bases in stroke aphasia

**DOI:** 10.1016/j.cortex.2022.07.014

**Published:** 2022-10

**Authors:** Ajay D. Halai, Blanca De Dios Perez, James D. Stefaniak, Matthew A. Lambon Ralph

**Affiliations:** a MRC Cognition & Brain Sciences Unit, University of Cambridge, Cambridge, CB2 7EF, United Kingdom; b Neuroscience and Aphasia Research Unit (NARU), School of Biological Sciences, The University of Manchester, Manchester, United Kingdom; c Division of Psychiatry and Applied Psychology, School of Medicine, University of Nottingham, Nottingham, United Kingdom

**Keywords:** Post-stroke aphasia, Assessment sensitivity, Comprehensive Aphasia, test, Principal component analysis

## Abstract

**Objective:**

Multi-assessment batteries are necessary for diagnosing and quantifying the multifaceted deficits observed post-stroke. Extensive batteries are thorough but impractically long for clinical settings or large-scale research studies. Clinically-targeted “shallow” batteries superficially cover a wide range of language skills relatively quickly but can struggle to identify mild deficits or quantify the impairment level. Our aim was to compare these batteries across a large group of chronic stroke aphasia and to test a novel data-driven reduced version of an extensive battery that maintained sensitivity to mild impairment, ability to grade deficits and the underlying component structure.

**Methods:**

We tested 75 chronic left-sided stroke participants, spanning global to mild aphasia. The underlying structure of these three batteries was analysed using cross-validation and principal component analysis, in addition to univariate and multivariate lesion-symptom mapping.

**Results:**

This revealed a four-factor solution for the extensive and data-reduced batteries, identifying phonology, semantic skills, fluency and executive function in contrast to a two-factor solution using the shallow battery (language severity and cognitive severity). Lesion symptom mapping using participants’ factor scores identified convergent neural structures for phonology (superior temporal gyrus), semantics (inferior temporal gyrus), speech fluency (precentral gyrus) and executive function (lateral occipitotemporal cortex). The two shallow battery components converged with the phonology and executive function clusters. In addition, we show that multivariate models could predict the component scores using neural data, however not for every component.

**Conclusions:**

Overall, the data-driven battery appears to be an effective way to save time yet retain maintained sensitivity to mild impairment, ability to grade deficits and the underlying component structure observed in post-stroke aphasia.

## Abbreviations

CAT: Comprehensive Aphasia Test
BDAE: Boston Diagnostic Aphasia Examination
WAB: Western Aphasia Battery
MTDDA: Minnesota Test for Differential Diagnosis of Aphasia
PICA: Porch Index of Communicative Ability
PSA: Post Stroke Aphasia
PCA: Principal Component Analysis
PALPA: Psycholinguistic Assessment of Language Processing in Aphasia
CCTp: Camel and Cactus pictures
BNT: Boston Naming Test
T: Tokens
WPM: Words Per Minute
MLU: Mean Length of Utterances
TTR: Type Token Ratio
VBCM: Voxel Based Correlational Methodology
PRoNTo: Pattern Recognition of Neuroimaging Toolbox
FWEc: Family Wise Error corrected
CSW: Comprehension Spoken Words
CWW: Comprehension Written Words
MCA: Middle Cerebral Artery

## 1 Introduction

Accurate and reliable ways to measure symptoms are critical for differential diagnosis and implementing optimal treatment pathways. For neuropsychological disorders, measuring symptoms is non-trivial for a number of reasons. First, patients have a wide range of deficits (e.g., memory, attention, speech and language, etc.), thus potentially necessitating a large assessment battery. Second, tests need sufficient dynamic range to capture all severities (complete impairment to well-recovered) with items varying in difficulty to avoid floor or ceiling effects. This is particularly important as deficits are graded along principal behavioural axes as opposed to falling into binary distinctions (Butler, Lambon Ralph, & Woollams, 2014; Lambon Ralph, Patterson, Graham, Dawson, & Hodges, 2003). Capturing this full range and severity of deficits requires an extensive, detailed assessment battery, which is rarely feasible in clinical settings, large-scale clinical trials or where patients have attention/fatigue deficits. The current study focuses on post stroke aphasia (PSA) as diagnosing language and cognitive deficits is particularly challenging due to considerable variation in the cognitive/language domains affected and the severity of the impairments. To save time, most batteries adopt a “shallow” approach, i.e., preserve the breadth (many domains) but reduce the test depth (number of items). In the current study, we directly compared an extensive battery (containing numerous tests each with many assessment items) against (a) a popular ‘shallow’ battery, the Comprehensive Aphasia Test (CAT) (Swinburn, Porter, & Howard, 2004); and (b) a novel data-driven ‘reduced’ test battery which limited the number of tests included but preserved their “depth”.

The long history of aphasia research contains many different approaches to assessment including early examples of systematic test batteries (Head, 1920). Many famous, popular batteries were designed to provide efficient clinical diagnoses of aphasia and its subtypes (i.e. Boston Diagnostic Aphasia Examination [BDAE] (Goodglass & Kaplan, 1972), Western Aphasia Battery [WAB] (Kertesz, 1982), Minnesota test for differential diagnosis of aphasia [MTDDA] (Schuell & Sefer, 1965), Porch Index of Communicative Ability [PICA] (Porch, 1967)). Many of these, however, have been found to be inadequate at identifying the nature of language impairments, and guiding future interventions and often lack reliability and validity (i.e., Byng, Kay, Edmundson, & Scott, 1990). Alternative approaches included batteries in the form of a ‘bank’ of psycholinguistically-sophisticated and detailed tests, such as the Psycholinguistic Assessment of Language Processing in Aphasia (Kay, Lesser, & Coltheart, 1992), from which experts select assessments to suit each individual patient. More recently, this collection of psycholinguistically-informed tests was transformed into a new ‘shallow’, systematic battery (the Comprehensive Aphasia Test (Howard, Swinburn, & Porter, 2010)) that has key advantages over its predecessors. The CAT was developed to: 1) diagnose impairments, 2) plan treatments, 3) guide follow up testing, 4) predict longitudinal (up to 12 months post onset) changes and 5) involve the patient in developing individualised goals (Bruce & Edmundson, 2010). The CAT is usually administered over 1–2 h and contains three sections: 1) cognitive screening; 2) language battery; and 3) a disability questionnaire. The language battery probes many different language activities each with a minimum number of carefully chosen items. The CAT was always intended to be an initial screening battery to be followed up by more detailed assessment of the identified areas of interest for each patient. Unsurprisingly, this efficient battery is used both clinically and in numerous research projects.

Inter-participant variations in PSA are well known. Binary aphasia classifications fail to capture important aspects of the underlying impairments and are unable to relate classifications and the underlying lesions (Basso, 2003; Howard et al., 2010; Poeck, 1983); indeed the authors of the CAT specifically noted that the tool was not developed to classify patients into subtypes. Based on detailed assessment batteries, contemporary studies have reconceptualised PSA in terms of graded variations along a limited number of underpinning principal language and cognitive dimensions (e.g., phonology, semantics, fluency and executive-cognitive skill), each of which is associated with specific brain regions (Butler et al., 2014; Halai, Woollams, & Lambon Ralph, 2017; Lacey, Skipper-Kallal, Xing, Fama, & Turkeltaub, 2017; Mirman, Chen, et al., 2015; Mirman, Zhang, Wang, Coslett, & Schwartz, 2015). Similar analyses have been conducted on each section of the CAT separately (Swinburn et al., 2004): one dimension was obtained for cognitive subtests and three factors for the language subtests (comprehension/writing, repetition and reading). The current project extended these analyses to test (a) if and when the latent structure of reduced batteries is the same as that derived from extended, in-depth batteries, and (b) if the latent structure remains the same when language and cognitive measures are analysed together.

The use of principal component analysis (PCA) and other data-reduction techniques are also relevant to the current study for another reason. From the task loadings, it is possible to identify which individual test best approximates each underlying dimension (Butler et al., 2014). We used this approach to generate a different kind of reduced battery. Specifically, principal component analysis was used to determine: 1) which subset of tests are the best proxies for each principal component; and 2) within each test, which subset of items best capture the variance in that test's data. Thus from an extensive battery, we generated a data-driven ‘reduced’ battery that is quick and efficient to administer, yet retains the extensive battery's underlying component structure.

Finally, we examined each battery's ability to identify the corresponding neural correlates. In previous work, we mapped the four principal components to the integrity of discrete brain regions (Halai et al., 2017; Halai, Woollams, & Lambon Ralph, 2018a) that align with results from fMRI language studies in healthy participants (Hickok & Poeppel, 2007; Price, 2012). Various studies have mapped different CAT subsets to brain damage (Hope et al., 2015; Hope, Leff, & Price, 2018; e.g., Hope, Seghier, Leff, & Price, 2013). To gain a complete picture, we compared neural correlates that arise from each of the three batteries. Lesion-symptom mapping can now be conducted using univariate or multivariate methods (Bates et al., 2003; DeMarco & Turkeltaub, 2018; Mah, Husain, Rees, & Nachev, 2014; Sperber & Karnath, 2018; Tyler, Marslen-Wilson, & Stamatakis, 2005; Zhang, Kimberg, Coslett, Schwartz, & Wang, 2014). Although there are strong advocates for each one, these alterative analyses tackle different fundamental questions, and have opposite strengths and weaknesses (Haufe et al., 2014; Hebart & Baker, 2018; Schumacher, Halai, & Lambon Ralph, 2019). Indeed, studies are beginning to report both results for transparency (i.e., Schumacher et al., 2019). Accordingly, we present both univariate and multivariate analyses for each test battery, with an adequate sample size based on previous investigations (e.g., Schumacher et al., 2019) and simulation studies (Sperber, Wiesen, & Karnath, 2019).

To summarise, the current study had the following aims and hypotheses. First, based on previous research we assume that the extended battery provides the most detailed aphasia profile (given the breadth and depth of the battery) and we used this as the “gold standard” comparator. Our first aim was to determine how sensitive the subtests of the CAT are for different aphasia deficits compared to equivalent subtests in the extended battery; we hypothesise that the former would be relatively insensitive to mild deficits compared to the latter given that the former contains fewer items. We note that the aim is not to assess the validity of the CAT subsets as has already been done in the original publication, but rather to determine if the limited dynamic range limits its ability to detect mild deficits. Second, we investigated how similar the underlying principal dimensions of the reduced batteries (CAT and novel battery) were to the dimensions of the extended battery (i.e., Halai et al., 2017); we hypothesise that the novel reduced battery would demonstrate similar latent structure as the extended battery, while the shallow battery would not due to the lack of dynamic range. Lastly, we utilised the principal dimensions from each battery to perform univariate and multivariate brain mapping, where we investigated how similar the reduced batteries were to the extended battery.

## 2 Materials and methods

We note that no part of the study procedures or analyses was pre-registered prior to the research being conducted. The data are not available in a public repository as we have ethical constraints (related to consent and confidentiality) to publicly release the data (ethics ref: MREC 01/08/94). Data are available upon completing a data sharing agreement (requests directed to MALR). We report how we determined our sample size, all data exclusions (if any), all inclusion/exclusion criteria, whether inclusion/exclusion criteria were established prior to data analysis, all manipulations, and all measures in the study.

### 2.1 Participants

Seventy-five chronic post-stroke (haemorrhagic or ischaemic) patients with aphasia (of any type or severity, spanning global to mild aphasia) were recruited. The sample size was not determined using a formal power calculation but was the largest sample that we could recruit at the time of this investigation. All participants were at least twelve months post-stroke, native English speakers with normal or corrected-to-normal hearing and vision. Exclusion criteria include: having more than one stroke, other neurological conditions, contraindications for MR scanning or being left handed premorbidly. Inclusion/exclusion criteria were fixed at the start of recruitment and before any data analysis was carried out. The demographic characteristics are presented in Supplementary Materials Section 1. In addition, thirty age matched controls were recruited and completed the reduced neuropsychological testing battery to obtain test cut-off scores. Informed consent was obtained from all participants prior to participation in the study under approval from the local ethics committee.

### 2.2 Neuropsychological *Assessment and cross-validation*

All participants were tested on an extensive neuropsychological battery (Butler et al., 2014; Halai et al., 2017). This battery assessed participants' language and cognitive abilities (described in Butler et al., 2014; Halai et al., 2017) across input/output phonology, semantic, executively-demanding and speech output tasks. Test materials that do not have copyright restrictions can be found at https://osf.io/aywmx/, the remaining tests can be obtained legally from the copyright holders in the cited references. These included subtests from the Psycholinguistic Assessments of Language Processing in Aphasia (PALPA) battery (Kay et al., 1992): auditory discrimination using non-word (PALPA 1) and word (PALPA 2) minimal pairs, and immediate and delayed repetition of non-words (PALPA 8) and words (PALPA 9). Tests from the 64-item Cambridge Semantic Battery (Bozeat, Lambon Ralph, Patterson, Garrard, & Hodges, 2000) were included: spoken and written versions of the word-to-picture matching task, the Camel and Cactus Test (pictures), and the picture-naming test. To increase the sensitivity to mild naming and semantic deficits, we used the Boston Naming Test (Kaplan, Goodglas, & Weintraub, 1983) and a written 96-trial synonym judgement test (Jefferies, Patterson, Jones, & Lambon Ralph, 2009). The spoken sentence comprehension task from the Comprehensive Aphasia Test (Swinburn, Baker, & Howard, 2005) was used to assess sentential receptive skills. The ‘Cookie theft’ description was used to assess speech production deficits. The coding procedure followed the method used by Borovsky, Saygin, Bates, and Dronkers (2007). Each utterance was marked and bound morphemes, repetitions, false starts, retraces, unintelligible material and interruptions were coded separately. Repeated and retraced utterances were excluded from analysis and only correct full utterances were coded. We applied the rule from Lee (1974) when determining the boundary of an utterance, where only one “and” conjunction per sentence was allowed when the “and” connected two independent clauses. From these transcriptions, we extracted the number of word tokens, type/token ratio (where types are unique items), mean length of utterances in morphemes and words per minute. The additional cognitive tests included forward and backward digit span (Wechsler, 1987), the Brixton Spatial Rule Anticipation Task (Burgess & Shallice, 1997), and Raven's Coloured Progressive Matrices (Raven, 1962). For the reduced battery we used the following tests where longer tests were reduced by half (see Supplementary Materials Section 2 for details): immediate word and nonword repetition, Cambridge and Boston naming tests, word-to-picture matching, synonym judgement, sentence comprehension, digit span, Raven's Coloured Progressive Matrices, Brixton spatial anticipation test and the four speech production measures from the Cookie Theft (tokens, type/token ratio, mean length of utterances in morphemes and words per minute). All participants also completed the short form of the Boston Diagnostic Aphasia Examination (Goodglass & Kaplan, 1972) to allow us to assign aphasia subtype labels to each patient (see Supplementary Materials Section 1 for details). We note that while the extended battery is detailed, it does not capture the full range of aphasia related deficits, specifically reading and writing (which was explored in a previous investigation; see Woollams, Halai, & Lambon Ralph, 2018).

All participants were part of a larger dataset collected at the University of Manchester over several years. We contacted all available participants to complete the CAT (electronic version) (Swinburn et al., 2004) omitting the disability questionnaire section as it was not relevant to the current study. The cognitive screen included a line bisection, semantic memory, word fluency, recognition memory, gesture object use, and arithmetic task. The language comprehension battery included written and spoken words, sentences and paragraphs. The expressive language battery had multiple tasks including repetition, naming, reading and writing. Each task included a range of typical words, complex words/objects, nonwords, and sentences. We used the picture description task to extract scores for appropriate/inappropriate information carrying words, syntactic variety, grammatical well-formedness and speed. All tests were scored according to the CAT scoring book and manual. Forty participants out of the original cohort were available for CAT testing (loss due to attrition).

In order to directly compare behavioural scores between batteries, we used normative cut-off scores. This meant that we could only directly compare (sub)tests that had normative data. We were able to identify cut-off boundaries for nine tests in the extended battery. These included digit span, repetition of words and non-words, spoken word comprehension, written word comprehension, semantic memory and picture naming (from normative data in Thompson et al., 2018) or original test manuals). In addition, as we created a new reduced battery (with fewer items than the original test), we collected control data from thirty age matched participants to determine cut-off scores (−2 S.D). We did this for: Cambridge and Boston picture naming, immediate word repetition, spoken word comprehension and synonym judgement tasks. We then matched subtests from the CAT to the tests noted above for pairwise comparison. For each pair, we calculate the percentage of patients within the normal and abnormal range, the correlation between the two batteries and proportion of patients that each battery ‘misclassified’ (i.e., within normal range of CAT test but impaired on extended battery and vice versa). In addition, we report summary demographic information (age, lesion volume and overall BDAE severity [0–5 scale, where 5 is minimal deficits/recovered] z-scored at the group level) for cases who were ‘mis-classified’ by the CAT for each test pairs.

We performed five-fold cross-validation analyses (Ballabio, 2015) (version 1.3 in MATLAB 2018a) to determine the optimal number of components for the PCA (see Halai, Woollams, & Lambon Ralph, 2020). The analyses determine what number of components provides the best solution corresponding to the lowest root mean squared error (N = number of tests). The behavioural data were scaled to percentage and the training data were normalised to z-scores before submitting to the cross-validation analysis. A secondary leave-one-out validation analysis was conducted on the optimum model to determine the generalisability of the solution (correlation between observed and predicted) (Abdi & Williams, 2010).

A varimax rotated PCA was conducted (SPSS v20.0) on the extensive battery for 75 patients, in order to determine the target structure. Test loadings were inspected for selected components (as defined by cross-validation analyses), where two tests were identified per component as representative proxies (high loading on the target dimension and near zero on others). In addition, we constrained selection with our knowledge of their clinical utility (i.e., practical and quicker assessments were favoured when multiple tests had high loadings). In addition, tests with more than 60 items were reduced by half to save on administration time (i.e., BNT and synonym judgement). We coded item level responses for 75 PSA participants for each test and performed an unrotated factor analysis restricted to a single factor. The top 50% of items loading most strongly on the identified factor were included in the reduced item set for each test. Tests with a particular internal structure (i.e., factorial design) were respected, such that factor analysis was conducted on each factorial level. Further details of the reduced tests are shown in Supplementary Materials Section 2.

### 2.3 Acquisition of neuroimaging data and processing

All participants underwent high resolution structural T1-weighted Magnetic Resonance Imaging (MRI) scans were acquired on a 3.0 T Philips Achieva scanner (Philips Healthcare, Best, The Netherlands) using an eight-element SENSE head coil. A T1-weighted inversion recovery sequence with 3D acquisition was employed, with the following parameters: TR (repetition time) = 9.0 ms, TE (echo time) = 3.93 ms, flip angle = 8°, 150 contiguous slices, slice thickness = 1 mm, acquired voxel size 1 × 1 × 1 mm³, matrix size 256 × 256, FOV = 256 mm, TI (inversion time) = 1150 ms, and SENSE acceleration factor 2.5 with a total scan acquisition time of 575 sec.

The neuroimaging images were processed with the automated lesion identification toolkit (ALI) (Seghier, Ramlackhansingh, Crinion, Leff, & Price, 2008) within Statistical Parametric Mapping software (SPM12: Wellcome Trust Centre for Neuroimaging, https://www.fil.ion.ucl.ac.uk/spm/). This performs a unified segmentation-normalisation procedure, which is optimised for focally lesioned brains.

We conducted univariate and multivariate brain-behaviour mapping using the PCA component scores derived from: 1) the extensive battery; 2) the data-driven reduced battery; and 3) the CAT. Both mapping approaches utilised images representing abnormalities in patients compared to a matched control population (hypo-intensity changes only). In the univariate analyses, we created three models (one for each PCA solution) and entered the corresponding components simultaneously. Voxel based correlational methodology (VBCM) (Tyler et al., 2005) was implemented in SPM12 to determine significant clusters, using a voxelwise threshold *p*<.001 with a family-wise error corrected (FWEc) clusterwise threshold *p*<.05. We computed models with and without lesion volume as an additional covariate. Estimated lesion volume was obtained through ALI. We used the pattern recognition of neuroimaging toolbox (PRoNTo v2.1; http://www.mlnl.cs.ucl.ac.uk/pronto/) (Schrouff et al., 2013) to determine whether individual scores on principal components could be predicted using multivariate analysis of the abnormality detected in the T1 image. Regression analysis used the relevance vector regression algorithm (Tipping, 2001) on a masked region defined by thresholding the lesion overlap map at 10%. This method is computationally efficient compared to other machines, which makes permutation testing of a large number of models more feasible. This implementation does not require hyperparameter optimisation and all models were assessed for performance using a leave-one-out cross-validation scheme (k-fold was not used due to small sample size). Model inference was determined by permutation testing (*N* = 1,000), where the dependant variable was shuffled randomly and the permuted correlations were used as the null distribution (alpha *p* < .05).

## 3 Results

### 3.1 Patient demographics and lesion overlap

**Fig. 1:**
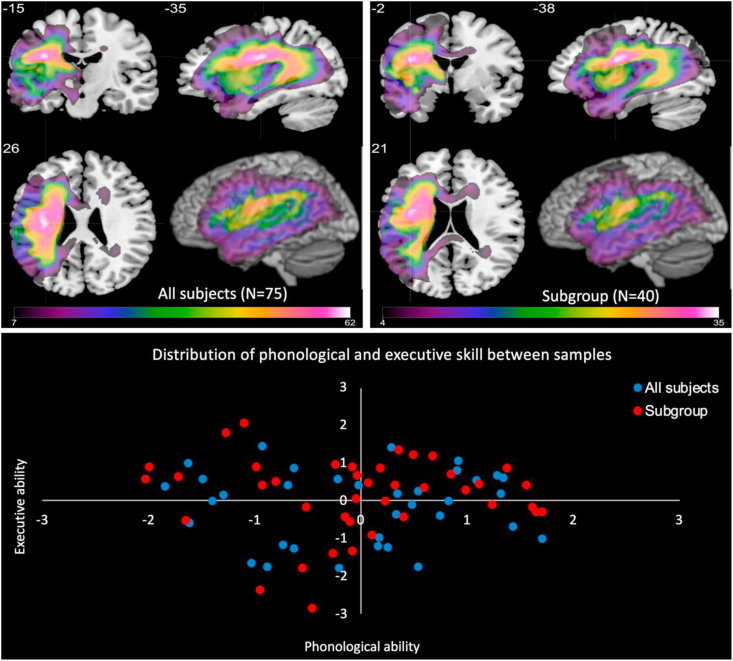
Lesion overlap map for all subjects (top left) and subgroup (top right) in MNI space. The lower panel shows the distribution of phonological and executive skill component scores for all subjects (blue and red combined) and subgroup (red only).

We show the lesion overlap maps for the participants in the study in Fig. 1. This Figure also shows the distribution of patient scores along two principal dimensions, for the full group and the subset that were available to complete the CAT testing. This shows that the subgroup were a representative subset of the full group (i.e., the covered all parts of the same multidimensional space). We found no difference between the full and subgroup participants for: age (62.59 [SD = 11.43] and 62.95 [SD = 11.56] years, respectively), education (12.04 [SD = 2.10] and 12.33 [SD = 2.37] years, respectively) and months post stroke (55.51 [SD = 48.22] and 52.08 [SD = 50.32] months, respectively). Participants ranged between 12 and 278 months post stroke. The gender composition of the groups was also not significantly different (55/20 and 27/13 males and females, respectively). The control and patient groups were matched for age (62.5 [SD = 5.48] and 62.59 [SD = 11.43] years, respectively).

We performed a Fisher exact test at each voxel across the brain to determine if the proportion of intact/damaged cases differed between the groups and found no significant differences (voxelwise *p*'s > .12), suggesting that the lesion profile was similar between groups. Furthermore, the lesion volume was not different between the full and subgroup (16809 [SD = 11555] and 16230 [SD = 11493] number of voxels, respectively). In terms of behavioural profiles, rather than compare all raw test scores we compared the principal component scores for the full and subgroups extracted from the largest dataset available. Again, we found no significant differences between groups for any component (*p*'s > .27).

### 3.2 Direct comparisons

We matched equivalent subtests from the CAT with the extensive and reduced battery (see Table 1 and Table 2 for pair-wise groupings, respectively). The Tables show direct comparisons between pair-wise CAT sub-tests and the equivalent test from the other two batteries. For each comparison, we show the R^2^ (variance explained) based on correlations, proportion of cases who were determined to have intact or impaired scores (using cut-off scores from norm data taken from the original test batteries, data extracted from Thompson et al., 2018, and using new data collected with age-matched control). The final two columns of the Tables indicate the proportion of patients who were identified as impaired on the two ‘detailed’ test batteries but not on the CAT (% missed with CAT) and those identified as impaired on the CAT but not on the two tests (% missed with Extensive/Reduced).

**Table 1:** Direct comparison of sensitivity between pair-wise CAT sub-tests and the equivalent extensive test. Each test indicates the proportion of cases determined as impaired/intact compared to control norms.

Neuropsychological tests		% Intact	% Impaired	R^2^	% missed with CAT	% missed with Extensive
Repetition	PALPA 9	12.50	87.50	.92	15	0
	CAT Word	27.50	72.50			
	PALPA 8	7.50	92.5	.67	20	0
	CAT Non-word	27.50	72.50			
Digit Span	Wechsler (forward)	17.50	82.50	.71	15	0
	CAT (forward)	32.50	67.50			
Naming	Cambridge Naming	10.00	90.00	.89	17.5	5
	CAT Naming	22.50	77.50			
	Boston Naming	5.00	95.00	.84	20	2.5
	CAT Naming	22.50	77.50			
Comprehension	Cambridge Spoken WPM	57.50	42.50	.44	17.5	5
	CAT spoken WPM	70.00	30.00			
	Cambridge Written WPM	57.50	42.50	.56	7.5	17.5
	CAT written WPM	47.50	52.50			
	96 Synonyms	15.00	85.00	.65	35	2.5
	CAT written WPM	47.50	52.50			
	96 Synonyms	15.00	85.00	.28	62.5	0
	CAT spoken WPM	77.50	22.50			
	Camel and Cactus (pictures)	47.50	52.50	.43	30	0
	CAT Semantic memory	77.50	22.50			

Abbreviations: Comprehension aphasia test (CAT), Psycholinguistic assessment of language processing in aphasia (PALPA) and word-to-picture matching (WPM).

**Table 2:** Direct comparison of sensitivity between pair-wise CAT sub-tests and the equivalent ‘reduced’ test battery (note: 50% fewer items compared to the extensive battery equivalent). Each test indicates the proportion of cases determined as impaired/intact compared to control norms.

Neuropsychological tests		% Intact	% Impaired	R^2^	% missed with CAT	% missed with Reduced
Repetition	PALPA 9	17.50	82.50	.91	12.5	2.5
	CAT Word	27.50	72.50			
Naming	Cambridge Naming	17.50	82.50	.90	15	10
	CAT Naming	22.50	77.50			
	Boston Naming	15.00	85.00	.87	10	2.5
	CAT Naming	22.50	77.50			
Comprehension	Cambridge Spoken WPM	52.50	47.50	.43	25	7.5
	CAT spoken WPM	70.00	30.00			
	96 Synonyms	15.00	85.00	.65	35	2.5
	CAT written WPM	47.50	52.50			
	96 Synonyms	15.00	85.00	.28	65	0
	CAT spoken WPM	77.50	22.50			

Abbreviations: Comprehension aphasia test (CAT), Psycholinguistic assessment of language processing in aphasia (PALPA) and word-to-picture matching (WPM).

**Fig. 2:**
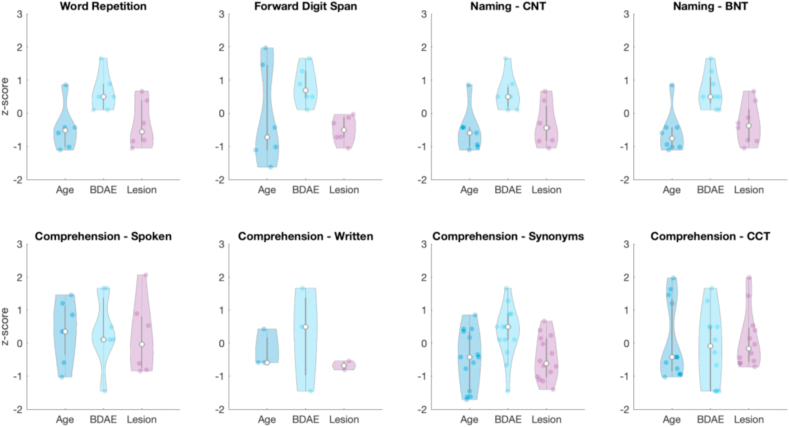
Demographic characteristics of patients misclassified by the CAT assessments with respect to the extended battery. The variables were converted to z-scores based on the full sample and a subset of misclassified patients are summarised for each test comparison. Overall, patients tended to be younger, have smaller lesions and have less severe BDAE diagnoses. Abbreviations: BDAE: Boston diagnostic aphasia examination; CNT: Cambridge naming test; BNT: Boston Naming test; CCT: camel and cactus test.

Each CAT subtest and its matched extensive test was found to be correlated, though varying across tests (R^2^ mean = .64, STD = .21, range = .28–.92), being best for repetition/naming and only moderate for semantics. The proportion of patients identified within the normal range by the CAT but impaired on the extensive test was significantly higher (mean = 24%, STD = 15.6%) than the reverse (mean = 3.25%, STD = 5.41%) (Wilcoxon rank test *p* = .0039). Fig. 2 reports characteristics of patients that were ‘misclassified’ by the CAT assessment with respect to the extended battery. Due to the relatively small number of deviant patients (3–14 across all comparisons) we only report the median value and no statistical test for significance was performed. We report z-scores based on the full sample such that any deviations can be interpreted with respect to the group average (i.e., z-score = 0). We observed a similar overall pattern across tests, where patients tend to have smaller lesions (median z-score range = −.68–−.03) and have less severe BDAE diagnosis (median z-score range = −.08 – .69). Patients also tended to be younger for all test pairs (except spoken word-to-picture matching, median = .35) with median z-score range between −.77 and −.42.

For completeness, we repeated this analysis by comparing the CAT subtests with their matched counterpart in the reduced battery; and, the results were similar to above. The correlation between tests was relatively high (R^2^ mean = .68, STD = .26, range = .31–.91), being best for repetition/naming and moderate for semantics. The proportion of patients identified within the normal range by the CAT but impaired on the reduced test was significantly higher (mean = 27%, STD = 20.8%) than the reverse (mean = 3.75%, STD = 4.1%) (Wilcoxon rank test *p* = .0313).

### 3.3 Identifying the underlying structure of language batteries

The k-fold analysis identified a four-factor solution for the extensive and reduced batteries for both the full patient cohort and the subgroup. Only a two-factor solution was identified for the CAT subgroup. Generalisability of the PCA models to the left-out cases was very high for all batteries and cohorts: extensive battery with all cases (*r* = .88) or subgroup (*r* = .88), reduced battery with all cases (*r* = .89) or subgroup (*r* = .90), and CAT with subgroup (*r* = .79).

**Fig. 3:**
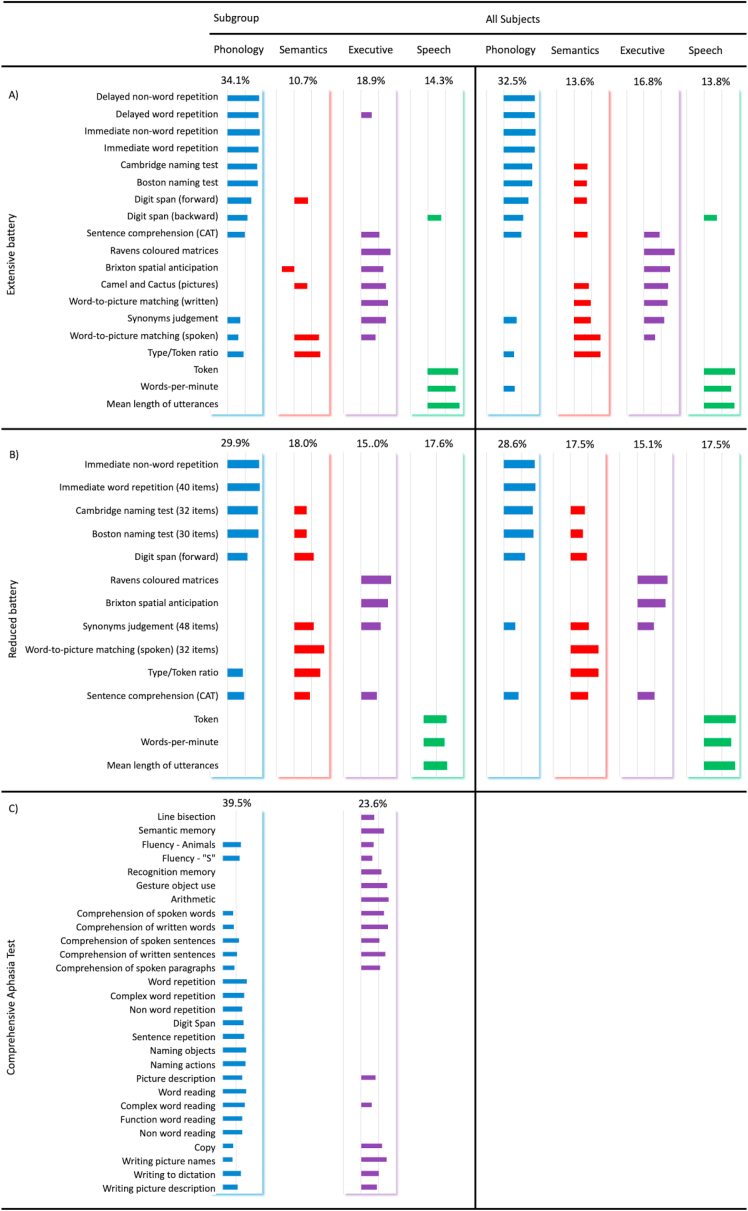
Composite figure showing test loadings for five principal component analyses: a) extensive battery on all cases and subgroup, b) reduced battery on all cases and subgroup, and c) comprehensive aphasia test on subgroup. Percent variance explained is indicated above each component column. Loadings between −.2 – .2 are omitted for clarity as they represent weak relationships to the components. The colour coding corresponds to each component: phonology (blue), semantics (red), executive (purple) and speech quanta (green).

Fig. 3 shows the factor loadings for each PCA solution. The PCA on the extensive battery with all cases replicated previous findings (Halai et al., 2017; 2018a): the four factors reflected phonological skill, executive function, speech quanta and semantics (accounting for 76.7% variance). The same component structure was found for all iterations of the extensive and reduced batteries (variance explained ranging between 77.9 and 80.4%), which was confirmed using correlational analyses (*r*'s > .95, *p*<.001). Therefore, regardless of sample size or battery used, the underlying PCA structure was stable and equivalent.

The results for the CAT were different, revealing a two-factor solution explaining 63.1% of the variance (see Fig. 3C). The first factor (39.5% variance) loaded onto tests requiring speech production and complex comprehension; termed language severity. The second factor (23.6% variance) included all other tests (language and non-language) which varied in difficulty (i.e., working memory demands and decision making), therefore was termed overall cognitive severity. The first CAT component was correlated with both the phonology (*r* = .88, *p* < .001) and semantics (*r* = .44, *p* < .005) dimensions derived from the extensive battery, while the second component correlated with the executive dimension (*r* = .79, *p*<.001). Finally, in order to make sure the type of tests included in the CAT were not a critical factor in shaping the underlying principal dimensions, we selected CAT subtests that closely reflected the types of tests included in the extended battery. This meant that we removed the following subtests from the CAT in a subsequent PCA: line bisection, gesture, arithmetic, copy and the writing tasks (naming, dictation and picture description). The results showed a consistent two-factor solution, similar to the solution with all tests (see Supplementary Materials section 5 for full details). We observed very high correlations between the corresponding factors between the two models where: *r* = .98 and *r* = .88 for components 1 and 2, respectively. This suggests that changing the inputs to the PCA does not influence the underlying latent structure of the CAT.

### 3.4 Mapping brain-behaviour relationships

**Fig. 4:**
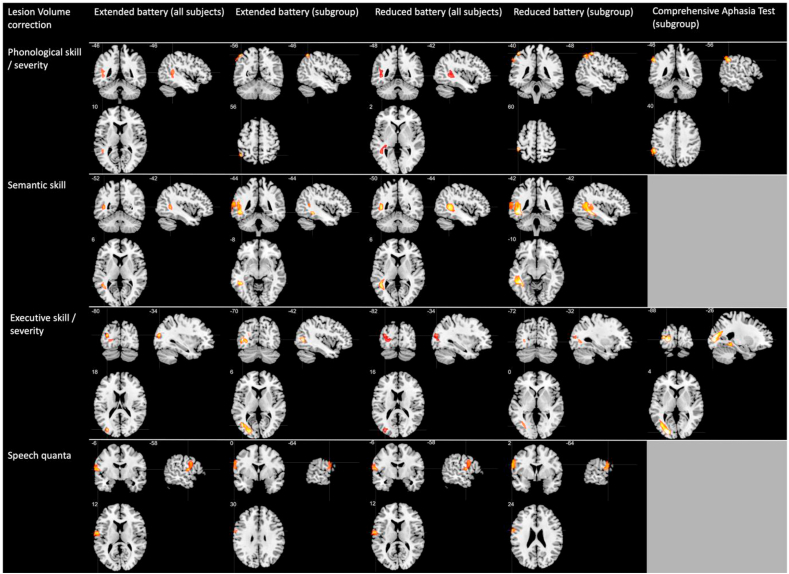
VBCM results for all components with lesion volume correction using voxelwise *p*<.001 and family wise error cluster correction *p*<.05 (except the phonology and executive skill for the reduced battery [all subjects], which is thresholded using a voxelwise *p* < .005 and family wise error cluster correction *p* < .09). The rows represent each principal component; phonological skill/severity, semantic skill, executive skill/severity and speech quanta. The grey patches in the final column indicate that there were no corresponding CAT components for semantic skill and speech quanta. Each panel has a cross hair located at the peak voxel. Scale t-values = 3–5.

Fig. 4 displays the univariate results showing significant clusters for every principal component (see Supplementary Materials Section 3 for peak information). For brevity, results with correction for lesion volume and demographic variables (age, education, months post onset and intracranial volume) are shown (additional analyses without lesion volume but with demographic variable correction are shown in Supplementary Materials Section 4). The results identified: 1) the superior temporal gyrus extending posteriorly into supramarginal gyrus and angular gyrus for phonology; 2) middle and ventral temporal cortex for semantics; and 3) precentral and inferior frontal gyrus for speech quanta. In addition, the lateral occipital cortex was identified for executive skills. This result was replicated across the extensive vs. data-driven reduced batteries and in the full cohort vs. patient subgroup. The only exception was that the phonology and executive component clusters survived correction at a lower threshold (voxel height *p* = .005, FWEc *p*<.09); however a direct comparison between the z-transformed whole brain maps revealed no difference between all two-way comparisons. The two CAT components identified clusters that overlapped with phonological and executive clusters from the extensive battery.

Table 3 shows the results from the multivariate analysis with and without lesion volume correction (cross-validated correlation coefficients). For models without lesion volume correction, the phonological component was predicted for the extensive battery (all and subgroup; cross validated *r* = .30 and .35, respectively) and the equivalent CAT component (cross validated *r* = .37). Results were not significant for the phonological skill component obtained from the reduced battery on the subgroup or in all cases. The semantic component was successfully predicted in all batteries (cross validated *r*'s > .43). The executive component was predicted for all batteries, including the equivalent CAT component (cross validated *r*'s > .30). The speech quanta component was predicted in the extensive and reduced battery for all cases (cross validated *r* = .25 and .28, respectively). For models with lesion volume correction, models significantly predicting semantic component scores were obtained for all batteries (cross validated *r*'s > .27). The executive component was predicted for the extensive and reduced subgroups (cross validated *r* = .49 and .36, respectively). The remaining models were not significant. In summary, the results for the extensive battery on all cases were very similar to the reduced battery for all cases (with and without lesion volume correction).

**Table 3:** Results from multivariate models predicting each principal component from brain abnormality images. The table shows the cross validated correlation between predicted and observed scores, where significant models were determined using permutation testing (*N* = 1,000). Model performances with and without lesion volume correction are shown.

Cross validated correlation		Extensive Battery (all cases)	Extensive Battery (subgroup)	Reduced Battery (all cases)	Reduced Battery (subgroup)	Comprehensive Aphasia Test (subgroup)
No lesion volume correction	Phonology	.30∗	.35∗	.22	.18	.37∗
	Semantics	.48∗	.48∗	.43∗	.63∗	
	Executive	.33∗	.59∗	.30∗	.40∗	.41∗
	Speech quanta	.25∗	.08	.28∗	.10	
Lesion volume correction	Phonology	.25	.29	.19	.12	.21
	Semantics	.37∗	.36∗	.27∗	.50∗	
	Executive	.21	.49∗	.19	.36∗	.20
	Speech quanta	.13	.004	.16	.03	

Footnote: ∗*p*<.05.

## 4 Discussion

Cognitive and language deficits due to brain injury or progressive disorders are typically multifaceted and can range from severe to very mild symptoms. There is a pressing need to detect neuropsychological deficits both effectively and efficiently across a wide range of severities and domains for clinical and research application. Most ‘shallow’ clinical batteries contain numerous tasks each with a small number of trials (typically <10). The limited dynamic range means these short subtests can be insensitive to mild impairments, fail to grade different levels of impairment and to detect longitudinal change. Such limitations are problematic in the clinic and research (e.g., missing mild impairments, inability to detect changing performance, detect graded dimensions, insufficient test score variance for correlation-based analyses such as lesion symptom mapping). Accordingly, the current study examined deficits in post-stroke aphasia by comparing an extensive, detailed test battery against a “shallow” assessment battery (the Comprehensive Aphasia Test; CAT) and a new, data-driven battery which preserved the depth but reduced the number of tasks.

Our results show that multiple subtests in the CAT were less sensitive to mild impairments than in the extensive battery (on average 24% cases missed) and the correlations between the tests, whilst good in general (average *R*^2^ = .64), varied (being best for repetition and weakest for semantics). Indeed, semantic/comprehension deficits were harder to detect in the CAT, with 7.5–62.5% of impaired cases missed. We also identified that the ‘misclassified’ cases by the CAT assessments with respect to the extended battery tended to have smaller lesions, have less severe BDAE diagnosis and be younger. This provides converging evidence that the CAT assessments may be insensitive to mild deficits, as all of these factors are typically associated with better outcomes. We found similar results with data from the reduced battery, where the CAT was less sensitive to mild impairments (on average 27% cases missed) and comprehension deficits harder to detect (25–65% impaired cases missed). These results are important as they highlight an apparent lack of sensitivity in the clinical screener that can impact patient diagnosis/treatment and/or research stratification. We note we were only able to compare four domains (repetition, naming, comprehension and digit span; see Table 1/2), despite the CAT spanning a broad range of test. This was due to the limitations of the data available in the extended battery and future research may focus on evaluating the sensitivity of the omitted subtests.; although we observed that the underlying structure of the CAT did not reflect independent domains as one might expect (see below).

Cross-validated PCA of the extensive battery revealed four, very robust dimensions of variation (phonology, semantics, fluency and cognitive-executive skill), which replicates previous findings (i.e., Halai et al., 2017, 2020). The CAT only generated two dimensions (phonology-language and generalised cognition) which, in the case of language, spanned two of the components derived from the extensive battery. In a previous examination, the authors of the CAT (Swinburn et al., 2004) reported three factors for the language assessments (comprehension/writing, repetition and reading) and one for the cognitive assessments. One key difference in methodology between the studies was that we conducted the PCA on all subtests of the CAT, while Swinburn et al., conducted two separate analyses (separating cognitive and language subtests); meaning that the contribution of executive/non-language functions were not controlled in the language domain. We have previously shown that failure to include executive/demanding non-verbal tests can lead to changes to the underlying latent factors (Halai, Woollams, & Lambon Ralph, 2018b), suggesting that one should include as much information about the syndrome as possible when performing decomposition analyses (see Alyahya, Halai, Conroy, & Lambon, 2020; Schumacher et al., 2019). As intended, the PCA data-driven reduced battery retained the four, robust language and cognitive components. Finally, in a series of univariate and multivariate lesion-symptom mapping analyses, the same pattern of results emerged; the extensive and data-driven reduced batteries revealed the same discrete areas associated with each of the four PCA components, whilst the CAT generated two areas of interest that overlapped with a subset of those observed from the alternative batteries.

It is, of course, important to consider the targets of investigation before selecting the most suitable assessments. The CAT was designed to provide a broad sampling of many language activities, which can be a critical clinical need. A complementary, orthogonal way to save time is to reduce the number of tests but preserve the test depth. The resulting larger dynamic range and sensitivity can be important in both the clinical and research; for example when needing to measure change over time (e.g., to track decline in progressive disorders, performance improvements in spontaneous recovery or after intervention, etc.) or when relating variation in language-cognitive performance to other factors and the distribution of underlying brain damage. The ability to fathom the underlying behavioural variations using PCA is also very likely to reflect the available dynamic range in the tests (like any correlation-based analysis, PCA requires sufficient variation). Whilst it can be important to assess performance on specific activities, the PCA on this large and diverse PSA cohort indicates that a large proportion of the total cohort variation (∼80%) can be captured by four orthogonal dimensions. This follows from the facts that (a) each task is not “pure” but instead reflects a combination of core language and cognitive skills and (b) that, resultantly, there is considerable collinearity across different tests (Butler et al., 2014; Halai et al., 2017; Patterson & Lambon Ralph, 1999). PCA also provides a data-driven solution to the question; which subset of tests should be selected from an extensive battery? It is also important to note that the CAT and reduced battery require approximately 90–120 min for administration in contrast to the extended battery, which takes approximately 6–10 h to complete. Of course, the two shortened tests might still be too long for some clinical situations. Indeed, even shorter batteries do exist such as the quick aphasia battery (Wilson, Eriksson, Schneck, & Lucanie, 2018), which can be administered in less than 30 min. Ultra-short batteries can be helpful in specific clinical/research situations, though it seems inescapable that the breadth and depth of information will be compromised. Ultimately, one cannot expect to measure everything in detail in no time at all. Instead, the goals of the assessment need to be considered.

Finally, we discuss the neural correlates and multivariate prediction results for the components scores across the different test batteries. The univariate VBCM analysis identified separable neural correlates for all component scores across all test batteries. The clusters were highly convergent with recent reports that have found: 1) phonology to be related to the supramarginal gyrus but extending into posterior superior temporal gyrus (Alyahya et al., 2020; Butler et al., 2014; Halai et al., 2017, 2018a; Hickok & Poeppel, 2007; Price, 2012); 2) semantics to be related to inferior and middle temporal gyrus (Lambon Ralph, Jefferies, Patterson, & Rogers, 2017); and 3) speech quanta being related to precentral gyrus extending into the insula (Halai et al., 2017; Kinoshita et al., 2015). The speech quanta cluster overlapped with the verbal quantity and motor speech clusters identified in Alyahya et al. (2020), which specifically focused on sub-dividing speech production into constituent parts (verbal quantity, verbal quality and motor speech).

The current study also identified regions in the left occipital, posterior temporal and posterior parietal lobe that were related to executive ability. There is evidence that the lateral temporo-occipital areas are activated for demanding visuo-spatial tasks and is commonly identified in explorations of the multiple demand network (Duncan, 2010; Fedorenko, Duncan, & Kanwisher, 2013; Humphreys & Lambon Ralph, 2017), which is required when completing the Raven's Coloured Progressive Matrices and Brixton Spatial Anticipation Test. In addition, recent investigations of the PSA population have found that executive ability is correlated with superior frontal and paracingulate regions (Alyahya, Halai, Conroy, & Lambon Ralph, 2018; Geranmayeh, Chau, Wise, Leech, & Hampshire, 2017; Lacey et al., 2017; Schumacher et al., 2019). Other similar studies have identified executive functions in the superior and middle frontal gyrus (Alyahya et al., 2020). Both results may reflect the likely effect of lesion size in the context of middle cerebral artery (MCA) stroke. Specifically, the occipital and superior frontal regions are only likely to be damaged if the MCA lesion is large (see Phan, Donnan, Wright, & Reutens, 2005 for lesion probability maps). This is confounded by the fact that large lesions are more likely to cause much more severe deficits overall; therefore, future studies could focus on these confounds in more detail and/or investigate stroke patients with posterior or anterior cerebral artery strokes.

The pattern of neural correlates across the components within different test batteries was remarkably similar. This probably reflects the fact that the batteries seem to assess the same four underlying dimensions. Even for the CAT, the lesion correlates for its two PCA components were almost identical to the clusters found for phonology and executive skills in the extensive battery, regardless whether all neuropsychological tests were included in the CAT analysis or a subset matching the detailed tests. The ability to predict the component scores using lesion information was also highly consistent when all cases were used in the extensive and reduced battery. The lesion data were able to predict all components without lesion volume correction (although the phonology component for reduced battery was at trend level). The results were less robust against chance for models with lesion volume correction, although the pattern of results between extended and reduced battery was the same across components. Results were mixed for the subgroup batteries, such that the models typically failed at predicting phonology and speech quanta. One reason for the lack of consistency might simply be due to the sample size, since multivariate decoding methodologies typically require large samples as data are partitioned into train/test sets for cross-validation. A recent simulation study (Sperber et al., 2019) suggested that approximately 100 subjects are required to have stable/reproducible beta parameter mapping, whereas for prediction of clinical outcomes the number peaked at 40 and was relatively stable from this point up to 100 cases. The numbers in the current study reflect these two ranges: 75 for the extensive battery (which generated robust results) and 40 for the subgroup analyses. As always, the current results will be improved through replication in future larger studies.

## Funding

This research was supported by grants from The Rosetrees Trust (no. A1699 to ADH and MALR), ERC (GAP: 670428 – BRAIN2MIND_NEUROCOMP to MALR), the 10.13039/501100000265Medical Research Council (MR/R023883/1 to MALR; MR/V031481/1 to ADH) and 10.13039/100010269Wellcome Trust (203914/Z/16/Z to JDS).

## Data availability

Behavioural and stimuli data are available in Supplementary Materials. Further data are available by request to the last author (MALR).

## Credit author statement

**Ajay D Halai:** Conceptualisation, Methodology, Formal Analysis, Writing – Review & Editing, Visualisation **Blanca De Dios Perez:** Formal Analysis, Investigation, Writing – Original Draft **James D Stefaniak:** Formal Analysis, Investigation, Writing – Review & Editing **Matthew A Lambon Ralph:** Conceptualisation, Writing – Review & Editing, Resources, Supervision, Funding Acquisition.

## Declaration of competing interest

The authors report no competing interests. The funders had no role in study design, data collection and analyses, decision to publish or preparation of the manuscript.
