# Supplementary material for: Efficient and effective assessment of deficits and their neural bases in stroke aphasia
Source: Cortex. 2022 Oct;155:333–46. doi: 10.1016/j.cortex.2022.07.014 (PMC9548407; doi:10.1016/j.cortex.2022.07.014)
Supplement: Multimedia component 1 [file mmc1.docx]

# Supplementary Materials

Section 1.

Table. Demographic information for the full sample of cases with chronic post stroke aphasia and the subgroup (indicated by ‘Months interval’ column). Cases are ordered by lesion volume. Abbreviations: Boston Diagnostic Aphasia Examination (BDAE); Transcortical sensory aphasia (TSA); Transcortical motor aphasia (TMA)

| ID | Gender | Aphasia classification | BDAE severity | Age at 1st testing | Years of education | Months post-stroke | Months interval | Lesion Vol |
| --- | --- | --- | --- | --- | --- | --- | --- | --- |
| 1 | F | Anomia | 5 | 43 | 16 | 15 | 25 | 175 |
| 2 | M | Anomia | 4 | 75 | 11 | 12 |  | 1481 |
| 3 | F | Anomia | 4.5 | 53 | 11 | 46 |  | 1526 |
| 4 | M | Anomia | 4.5 | 68 | 11 | 21 | 34 | 3311 |
| 5 | M | Broca | 1.5 | 61 | 11 | 15 |  | 3528 |
| 6 | F | Conduction | 2 | 46 | 16 | 21 | 29 | 3897 |
| 7 | M | Anomia | 4 | 50 | 19 | 15 | 14 | 4538 |
| 8 | M | Conduction | 3 | 68 | 11 | 38 |  | 4773 |
| 9 | M | Anomia | 4 | 65 | 17 | 25 |  | 4806 |
| 10 | M | Conduction | 3 | 67 | 11 | 13 | 20 | 4879 |
| 11 | F | Broca | 4 | 48 | 12 | 16 |  | 5273 |
| 12 | M | TSA | 3.5 | 63 | 12 | 24 |  | 5822 |
| 13 | M | Conduction | 3 | 67 | 17 | 12 | 20 | 6557 |
| 14 | M | Anomia | 3.5 | 65 | 10 | 85 | 11 | 6607 |
| 15 | F | Wernicke | 3 | 77 | 16 | 33 |  | 6843 |
| 16 | M | Anomia | 5 | 56 | 16 | 17 | 15 | 6974 |
| 17 | F | Anomia | 3.5 | 51 | 11 | 65 | 34 | 6975 |
| 18 | M | Anomia | 4 | 84 | 9 | 35 |  | 7854 |
| 19 | F | Anomia | 3.5 | 68 | 16 | 22 |  | 8118 |
| 20 | M | Anomia | 3.5 | 87 | 12 | 35 |  | 8238 |
| 21 | M | Anomia | 3.5 | 44 | 11 | 39 | 34 | 8437 |
| 22 | M | Anomia | 3.5 | 86 | 9 | 17 | 21 | 8528 |
| 23 | M | Mixed Non-fluent | 3.5 | 68 | 11 | 14 | 83 | 8788 |
| 24 | F | Anomia | 3.5 | 73 | 11 | 88 |  | 8921 |
| 25 | F | Anomia | 3.5 | 69 | 19 | 39 | 33 | 9159 |
| 26 | F | Global | 1 | 77 | 11 | 18 | 4 | 9229 |
| 27 | F | Anomia | 4.5 | 52 | 12 | 75 | 68 | 9767 |
| 28 | F | Global | 1 | 56 | 11 | 29 | 2 | 10051 |
| 29 | M | Anomia | 4 | 67 | 11 | 59 |  | 10073 |
| 30 | M | TMA | 3.5 | 76 | 11 | 115 |  | 11239 |
| 31 | M | Broca | 1 | 85 | 10 | 45 |  | 11393 |
| 32 | M | Broca | 3.5 | 52 | 17 | 33 | 84 | 11915 |
| 33 | F | Mixed Non-fluent | 2 | 75 | 11 | 158 | 23 | 12057 |
| 34 | M | Broca | 2 | 82 | 10 | 14 | 28 | 12131 |
| 35 | F | Anomia | 3 | 58 | 11 | 278 | 23 | 12699 |
| 36 | M | Broca | 2 | 59 | 13 | 37 | 34 | 13080 |
| 37 | M | Global | 1 | 78 | 11 | 12 | 4 | 13187 |
| 38 | F | Anomia | 3 | 77 | 11 | 55 | 34 | 13577 |
| 39 | M | Mixed Non-fluent | 1 | 58 | 13 | 31 |  | 14625 |
| 40 | M | Anomia | 4.5 | 51 | 11 | 41 | 2 | 14681 |
| 41 | M | Global | 1 | 66 | 11 | 12 |  | 14890 |
| 42 | M | Anomia | 3.5 | 66 | 11 | 124 |  | 15492 |
| 43 | M | anomia | 5 | 80 | 11 | 77 | 3 | 15857 |
| 44 | M | Anomia | 4 | 59 | 11 | 34 | 33 | 16433 |
| 45 | M | Broca | 2 | 80 | 12 | 65 |  | 18163 |
| 46 | M | Broca | 3.5 | 58 | 11 | 134 | 69 | 18392 |
| 47 | M | Broca | 2.5 | 54 | 13 | 36 | 33 | 18632 |
| 48 | M | Anomia | 4.5 | 63 | 12 | 12 |  | 18639 |
| 49 | F | Anomia | 4 | 44 | 13 | 37 | 25 | 18948 |
| 50 | M | Global | 1 | 74 | 11 | 18 |  | 19500 |
| 51 | M | Broca | 1.5 | 51 | 12 | 34 |  | 20043 |
| 52 | M | Anomia | 3.5 | 85 | 10 | 68 |  | 21489 |
| 53 | M | Mixed Non-fluent | 1 | 73 | 11 | 23 | 37 | 22732 |
| 54 | M | Anomia | 3.5 | 51 | 13 | 73 | 30 | 22948 |
| 55 | F | TMA | 3 | 73 | 11 | 47 | 25 | 23863 |
| 56 | M | Mixed Non-fluent | 3.5 | 67 | 11 | 119 |  | 26097 |
| 57 | M | Broca | 2.5 | 50 | 12 | 16 |  | 26218 |
| 58 | F | Mixed Non-fluent | 4 | 67 | 14 | 173 |  | 26283 |
| 59 | F | Mixed Non-fluent | 3 | 67 | 11 | 58 | 3 | 26491 |
| 60 | M | Global | 1 | 72 | 11 | 41 |  | 27054 |
| 61 | M | Broca | 2 | 62 | 11 | 103 |  | 27242 |
| 62 | M | Mixed Non-fluent | 1.5 | 81 | 11 | 68 |  | 28144 |
| 63 | M | Mixed Non-fluent | 1 | 67 | 11 | 44 |  | 31317 |
| 64 | M | Mixed Non-fluent | 1 | 63 | 12 | 41 |  | 31599 |
| 65 | M | Global | 1 | 72 | 11 | 153 |  | 32981 |
| 66 | M | Global | 1 | 58 | 13 | 57 | 31 | 33239 |
| 67 | M | Mixed Non-fluent | 2 | 64 | 11 | 29 | 25 | 33239 |
| 68 | M | Mixed Non-fluent | 2 | 79 | 11 | 63 |  | 33678 |
| 69 | M | Mixed Non-fluent | 1 | 78 | 13 | 36 | 34 | 34242 |
| 70 | M | Broca | 3 | 73 | 11 | 114 | 22 | 36877 |
| 71 | M | Global | 1 | 52 | 11 | 73 | 23 | 37822 |
| 72 | M | Mixed Non-fluent | 1 | 69 | 11 | 83 | 4 | 37850 |
| 73 | F | Mixed Non-fluent | 1 | 52 | 11 | 98 |  | 40313 |
| 74 | M | Global | 1 | 68 | 12 | 49 | 71 | 41379 |
| 75 | M | Mixed Non-fluent | 3 | 76 | 11 | 189 |  | 42568 |

Section 2.

In the following section we show the results of the factor analyses (unrotated single dimension) performed on the individual item level scores of each test (or their condition manipulations). This identified each items’ loading onto the factor explaining the largest amount of variance in the data; the top 50% of items were included. Table 2 shows the items that were included in the reduced tests for: 1) PALPA 9 (word repetition), 2) Boston naming test (BNT), 3) Cambridge semantic battery 64-item picture naming, and 4) 96-item synonym judgement test. Each column in Table 2 shows the top 50% loading items following the factor analysis, with the bottom section showing descriptive statistics of the loading values.

The PALPA 9 test for word repetition consists of 80 items and a one factor solution explained 48.87% of the variance. The BNT has 60 items and a one factor solution explained 39.39% of the variance. As the same Cambridge semantic battery items were used in both the picture naming and word-picture matching tests, we wanted to ensure item consistency across tests. Picture naming had a larger variance of scores in the stroke cohort compared to the spoken word to picture matching test (SD = 33.9 and 11.5, respectively) and so we performed the factor analysis on the Cambridge naming test (CNT). The battery consists of 64 items with two animacy groups (living and non-living). A factor analysis on each dimension showed that the model for the living category explained 44.05% variance and the non-living model explained 46.74% variance. The same reduced item list derived from the CNT test data was used in the reduced spoken word-to-picture matching test. The 96-synonym judgement test is split into six groups along high/low frequency (HF/LF) and high/mid/low imageability (HI/MI/LI) dimensions. A factor analysis on the item scores within each group produced the following models: HF HI 33.50% variance explained; HF MI 26.34% variance explained; HF LI 16.70% variance explained; LF HI 41.80% variance explained; LF MI 24.99% variance explained; LF LI 14.76% variance explained.

Table. Item list for the reduced tests: 1) psycholinguistic assessment of language processing in aphasia - word repetition (PALPA 9), 2) Boston naming, 3) Cambridge semantic battery 64-item picture naming, and 4) Synonym judgement. Each column is the result of a separate factor analysis and descriptive statistics for the loading of the items are shown at the bottom. Abbreviations: High frequency (HF), low frequency (LF), high imageability (HI), mid imageability (MI), low imageability (LI).

|  | PALPA 9 | Boston Naming Test | Cambridge Naming Test | | Synonym Judgement | | | | | |
| --- | --- | --- | --- | --- | --- | --- | --- | --- | --- | --- |
|  |  |  | Living | Non-Living | HF-HI | HF-MI | HF-LI | LF-HI | LF-MI | LF-LI |
|  | Village | Scissors | Camel | Scissors | Forest | Pattern | Value | Tulip | Humour | Alias |
|  | Marriage | Mushroom | Swan | Plane | Student | Freedom | Function | Frog | Expanse | Despot |
|  | Hospital | Pencil | Owl | Candle | River | Ancient | Reason | Puppy | Quake | Protocol |
|  | Tractor | Hanger | Apple | Piano | Money | Master | Tendency | Zipper | Rogue | Dirge |
|  | Moment | Dart | Cat | Saw | Sun | Property | Advantage | Lobster | Enamel | Audit |
|  | Potato | Racquet | Cow | Toaster | Plant | Fashion | Keep | Jewel | Boredom | Deity |
|  | Character | Harmonica | Pear | Train | Rock | Distance | Basic | Kitten | Gallant | Arbiter |
|  | Audience | Canoe | Banana | Brush | Child | Pair | Effect | Chestnut | Adultery | Bequest |
|  | Gravy | Volcano | Rhino | Basket |  |  |  |  |  |  |
|  | Battle | Mask | Pineapple | Key |  |  |  |  |  |  |
|  | Window | Igloo | Horse | Comb |  |  |  |  |  |  |
|  | Fire | Unicorn | Dog | Bus |  |  |  |  |  |  |
|  | Hotel | Comb | Elephant | Dustbin |  |  |  |  |  |  |
|  | Quality | Seahorse | Mouse | Helicopter |  |  |  |  |  |  |
|  | Treason | Noose | Strawberry | Envelope |  |  |  |  |  |  |
|  | Miracle | Whistle | Frog | Lorry |  |  |  |  |  |  |
|  | System | House |  |  |  |  |  |  |  |  |
|  | Gravity | Funnel |  |  |  |  |  |  |  |  |
|  | Radio | Helicopter |  |  |  |  |  |  |  |  |
|  | Member | Saw |  |  |  |  |  |  |  |  |
|  | Idea | Bench |  |  |  |  |  |  |  |  |
|  | Bonus | Harp |  |  |  |  |  |  |  |  |
|  | Coffee | Camel |  |  |  |  |  |  |  |  |
|  | Mother | Toothbrush |  |  |  |  |  |  |  |  |
|  | Concept | Dominos |  |  |  |  |  |  |  |  |
|  | Student | Scroll |  |  |  |  |  |  |  |  |
|  | Alcohol | Bed |  |  |  |  |  |  |  |  |
|  | Picture | Snail |  |  |  |  |  |  |  |  |
|  | Attitude | Acorn |  |  |  |  |  |  |  |  |
|  | Manner | Tree |  |  |  |  |  |  |  |  |
|  | Church |  |  |  |  |  |  |  |  |  |
|  | Dogma |  |  |  |  |  |  |  |  |  |
|  | Effort |  |  |  |  |  |  |  |  |  |
|  | Elephant |  |  |  |  |  |  |  |  |  |
|  | Crisis |  |  |  |  |  |  |  |  |  |
|  | Spider |  |  |  |  |  |  |  |  |  |
|  | Onion |  |  |  |  |  |  |  |  |  |
|  | Purpose |  |  |  |  |  |  |  |  |  |
|  | Funnel |  |  |  |  |  |  |  |  |  |
|  | Tribute |  |  |  |  |  |  |  |  |  |
| Loadings |  |  |  |  |  |  |  |  |  |  |
| Mean | 0.754 | 0.718 | 0.727 | 0.744 | 0.677 | 0.599 | 0.490 | 0.705 | 0.582 | 0.472 |
| SD | 0.029 | 0.048 | 0.042 | 0.049 | 0.079 | 0.065 | 0.090 | 0.038 | 0.065 | 0.124 |
| Min | 0.704 | 0.629 | 0.680 | 0.688 | 0.563 | 0.533 | 0.416 | 0.675 | 0.480 | 0.354 |
| Max | 0.822 | 0.810 | 0.842 | 0.875 | 0.763 | 0.743 | 0.692 | 0.795 | 0.676 | 0.706 |

Section 3 .

Table. Neural correlate peaks for behavioural component scores after accounting for lesion volume, age, years in education, months post onset and intracranial volume.

| Battery | Component | Cluster (no. of voxels) | Anatomy | Side | Z | MNI co-ordinates | | |
| --- | --- | --- | --- | --- | --- | --- | --- | --- |
|  |  |  |  |  |  | x | y | z |
| Extended (all cases) | Phonology | 186 | Supramarginal gyrus posterior | Left | 3.58 | -46 | -46 | 10 |
|  |  |  | Left Superior longitudinal fasciculus | Left | 3.53 | -44 | -44 | 2 |
|  |  |  | Left Inferior frontal occipital fasciculus | Left | 3.4 | -34 | -32 | 2 |
|  |  |  | Planum temporale | Left | 3.27 | -30 | -32 | 16 |
|  | Executive | 237 | Lateral occipital cortex superior | Left | 4.35 | -34 | -80 | 18 |
|  |  |  | Forceps major |  | 4.03 | -22 | -84 | 6 |
|  |  |  | Left Inferior frontal occipital fasciculus | Left | 3.19 | -30 | -78 | 0 |
|  | Speech quanta | 450 | Central operculum cortex | Left | 3.99 | -58 | -6 | 12 |
|  |  |  | Precentral gyrus | Left | 3.79 | -64 | 0 | 28 |
|  |  |  | Precentral gyrus | Left | 3.58 | -60 | 0 | 18 |
|  | Semantics | 230 | Left Superior longitudinal fasciculus | Left | 3.94 | -42 | -52 | 6 |
|  |  |  | Left Inferior longitudinal fasciculus | Left | 3.51 | -30 | -74 | 10 |
|  |  |  | Left Inferior longitudinal fasciculus | Left | 3.29 | -36 | -62 | 10 |
| Extended (subgroup) | Phonology | 283 | Angular gyrus | Left | 3.99 | -46 | -56 | 56 |
|  |  |  | Supramarginal gyrus posterior | Left | 3.94 | -56 | -46 | 42 |
|  |  |  | Supramarginal gyrus posterior | Left | 3.94 | -60 | -54 | 44 |
|  |  |  | Supramarginal gyrus posterior | Left | 3.44 | -64 | -52 | 36 |
|  | Executive | 846 | Lateral occipital cortex inferior | Left | 4.56 | -42 | -70 | 6 |
|  |  |  | Lateral occipital cortex superior | Left | 4.04 | -34 | -82 | 14 |
|  |  |  | Forceps major |  | 4.04 | -24 | -84 | 6 |
|  |  |  | Left Inferior longitudinal fasciculus | Left | 3.74 | -32 | -74 | 2 |
|  |  | 147 | Precentral gyrus | Right | 4.04 | 24 | -20 | 60 |
|  |  |  | Right Corticospinal tract | Right | 3.8 | 18 | -28 | 54 |
|  |  |  | Precentral gyrus | Right | 3.72 | 30 | -22 | 44 |
|  |  |  | Postcentral gyrus | Right | 3.21 | 32 | -26 | 52 |
|  | Speech quanta | 157 | Precentral gyrus | Left | 3.5 | -64 | 0 | 30 |
|  |  |  | Precentral gyrus | Left | 3.48 | -60 | 0 | 18 |
|  |  |  | Central operculum cortex | Left | 3.32 | -54 | -10 | 10 |
|  |  |  | Precentral gyrus | Left | 3.22 | -66 | -4 | 14 |
|  | Semantics | 156 | Frontal orbital cortex | Left | 4.34 | -20 | 16 | -28 |
|  |  |  | Frontal orbital cortex | Left | 3.96 | -30 | 26 | -26 |
|  |  | 1161 | Left Inferior longitudinal fasciculus | Left | 4.04 | -44 | -44 | -8 |
|  |  |  | Middle temporal gyrus temocc | Left | 3.89 | -46 | -50 | 10 |
|  |  |  | Left Superior longitudinal fasciculus | Left | 3.86 | -52 | -44 | -6 |
|  |  |  | Supramarginal gyrus posterior | Left | 3.8 | -52 | -46 | 18 |
| Reduced (all cases) | Phonology | 380 | Left Superior longitudinal fasciculus | Left | 3.26 | -42 | -48 | 2 |
|  |  |  | Supramarginal gyrus posterior | Left | 3.23 | -46 | -46 | 10 |
|  |  |  | Left Inferior frontal occipital fasciculus | Left | 3.11 | -32 | -32 | 4 |
|  |  |  | Planum temporale | Left | 2.97 | -30 | -32 | 16 |
|  | Executive | 354 | Lateral occipital cortex superior | Left | 3.47 | -34 | -82 | 16 |
|  |  |  | Forceps major |  | 3.28 | -20 | -86 | 6 |
|  |  |  | Lateral occipital cortex inferior | Left | 3.27 | -28 | -84 | 6 |
|  |  |  | Left Inferior frontal occipital fasciculus | Left | 2.93 | -30 | -78 | 0 |
|  | Speech quanta | 421 | Central operculum cortex | Left | 3.88 | -58 | -6 | 12 |
|  |  |  | Precentral gyrus | Left | 3.73 | -64 | 0 | 28 |
|  |  |  | Precentral gyrus | Left | 3.5 | -60 | 0 | 18 |
|  | Semantics | 525 | Middle temporal gyrus tempocci | Left | 4.36 | -44 | -50 | 6 |
|  |  |  | Supracalcarine cortex | Left | 3.47 | -24 | -64 | 22 |
|  |  |  | Forceps major |  | 3.4 | -28 | -74 | 12 |
|  |  |  | Lateral occipital cortex superior | Left | 3.27 | -34 | -76 | 18 |
| Reduced (subgroup) | Phonology | 407 | Postcentral gyrus | Left | 4.36 | -48 | -40 | 60 |
|  |  |  | Supramarginal gyrus posterior | Left | 4.24 | -56 | -46 | 42 |
|  |  |  | Supramarginal gyrus posterior | Left | 3.99 | -60 | -54 | 44 |
|  |  |  | Angular gyrus | Left | 3.92 | -46 | -56 | 56 |
|  | Executive | 164 | Precentral gyrus | Right | 4.14 | 24 | -20 | 60 |
|  |  |  | Right Corticospinal tract | Right | 3.87 | 20 | -28 | 50 |
|  |  |  | Right Corticospinal tract | Right | 3.71 | 16 | -26 | 58 |
|  |  |  | Precentral gyrus | Right | 3.54 | 30 | -22 | 44 |
|  |  | 182 | Left Inferior longitudinal fasciculus | Left | 3.48 | -32 | -72 | 0 |
|  |  |  | Left Inferior longitudinal fasciculus | Left | 3.45 | -38 | -62 | 0 |
|  |  |  | Lateral occipital cortex inferior | Left | 3.42 | -26 | -84 | 6 |
|  |  |  | Lateral occipital cortex superior | Left | 3.32 | -34 | -82 | 16 |
|  | Speech quanta | 308 | Precentral gyrus | Left | 3.71 | -64 | 2 | 24 |
|  |  |  | Precentral gyrus | Left | 3.67 | -62 | 0 | 34 |
|  |  |  | Precentral gyrus | Left | 3.51 | -60 | -4 | 14 |
|  |  |  | Precentral gyrus | Left | 3.4 | -56 | 4 | 26 |
|  | Semantics | 2566 | Left Inferior longitudinal fasciculus | Left | 5.13 | -42 | -42 | -10 |
|  |  |  | Middle temporal gyrus tempocci | Left | 4.44 | -46 | -50 | 10 |
|  |  |  | Left Inferior longitudinal fasciculus | Left | 4.24 | -36 | -50 | -10 |
|  |  |  | Middle temporal gyrus tempocci | Left | 4.2 | -52 | -44 | -4 |
| CAT - all subtests (subgroup) | Phonological severity | 325 | Supramarginal gyrus posterior | Left | 4.16 | -56 | -46 | 40 |
|  |  |  | Supramarginal gyrus posterior | Left | 4.12 | -60 | -54 | 44 |
|  |  |  | Supramarginal gyrus posterior | Left | 3.75 | -64 | -52 | 36 |
|  |  |  | Lateral occipital cortex superior | Left | 3.59 | -48 | -60 | 54 |
|  | Executive severity | 207 | Lingual gyrus | Left | 5.13 | -20 | -56 | -4 |
|  |  |  | Left Hippocampus | Left | 4.42 | -30 | -40 | -6 |
|  |  | 165 | Precentral gyrus | Right | 4.86 | 24 | -18 | 54 |
|  |  |  | Right Corticospinal tract | Right | 4.32 | 22 | -28 | 48 |
|  |  | 1439 | Lateral occipital cortex inferior | Left | 4.84 | -26 | -86 | 4 |
|  |  |  | Lateral occipital cortex superior | Left | 4.7 | -28 | -84 | 12 |
|  |  |  | Lateral occipital cortex inferior | Left | 4.47 | -40 | -74 | 12 |
|  |  |  | Lateral occipital cortex superior | Left | 4.21 | -32 | -76 | 12 |
|  |  | 185 | Precuneous cortex | Right | 4.23 | 6 | -60 | 16 |
|  |  |  | Cuneal cortex | Right | 4.11 | 8 | -68 | 22 |
|  |  |  | Precuneous cortex | Right | 4.05 | 8 | -54 | 24 |
|  |  |  | Precuneous cortex | Right | 3.52 | 18 | -66 | 22 |
|  |  | 148 | Right Hippocampus | Right | 3.84 | 28 | -10 | -18 |
| CAT - partial subtests (subgroup) | Phonological severity | 354 | Supramarginal gyrus posterior | Left | 4.21 | -56 | -44 | 40 |
|  |  |  | Supramarginal gyrus posterior | Left | 4.12 | -60 | -54 | 44 |
|  |  |  | Supramarginal gyrus posterior | Left | 3.84 | -64 | -52 | 36 |
|  |  |  | Lateral occipital cortex superior | Left | 3.67 | -48 | -60 | 54 |
|  |  | 158 | Left Superior longitudinal fasciculus | Left | 3.61 | -38 | -50 | 18 |
|  |  |  | Supramarginal gyrus posterior | Left | 3.45 | -48 | -48 | 34 |
|  |  |  | Supramarginal gyrus posterior | Left | 3.43 | -46 | -46 | 10 |
|  |  |  | Left Superior longitudinal fasciculus | Left | 3.38 | -38 | -42 | 14 |
|  | Executive severity | 1607 | Lateral occipital cortex superior | Left | 4.94 | -32 | -88 | 12 |
|  |  |  | Lateral occipital cortex inferior | Left | 4.73 | -40 | -76 | 12 |
|  |  |  | Left Inferior frontal occipital fasciculus | Left | 4.25 | -30 | -80 | -2 |
|  |  |  | Lateral occipital cortex superior | Left | 4.17 | -40 | -66 | 18 |
|  |  | 148 | Precuneous cortex | Right | 4.29 | 6 | -60 | 14 |
|  |  |  | Precuneous cortex | Right | 3.7 | 8 | -74 | 34 |

Section 4.


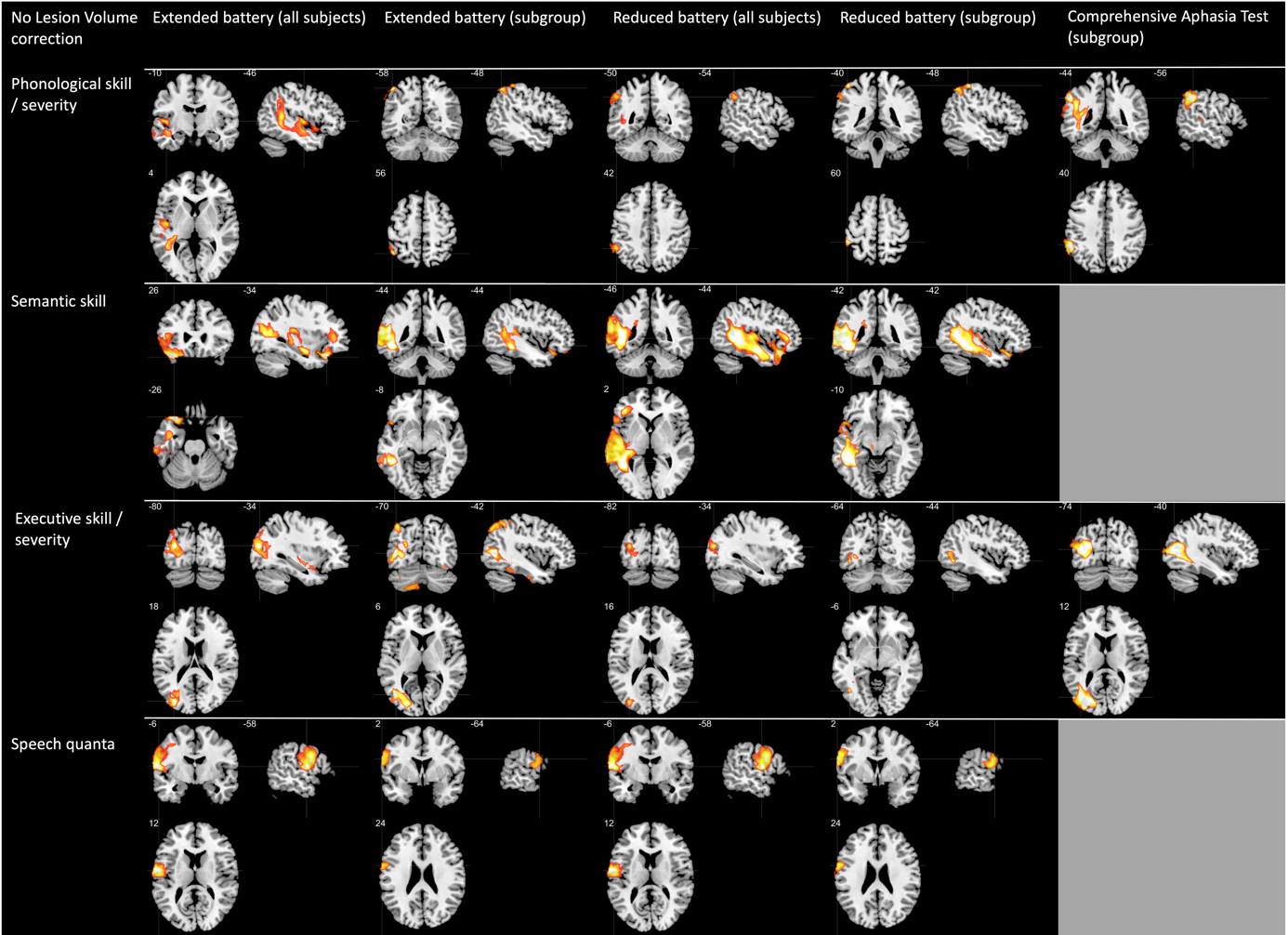


Figure. VBCM results for all principal components for each test battery. The components for each column were entered simultaneously and with the following covariates: age, years in education, months post onset, and intracranial volume. The results are thresholded using p < .001 voxelwise with family wise error cluster correction p < .05. The rows represent each principal component; phonological skill / severity, semantic skill, executive skill/severity and speech quanta. The grey patches in the final column indicate that there were no corresponding CAT components for semantic skill and speech quanta.

Table. Neural correlate peaks for behavioural component scores after accounting for age, years in education, months post onset and intracranial volume.

| Battery | Component | Cluster (no. of voxels) | Anatomy | Side | Z | MNI co-ordinates | | |
| --- | --- | --- | --- | --- | --- | --- | --- | --- |
|  |  |  |  |  |  | x | y | z |
| Extended (all cases) | Phonology | 2646 | Heschls gyrus | Left | 4.16 | -46 | -10 | 4 |
|  |  |  | Supramarginal gyrus posterior | Left | 4.06 | -54 | -50 | 42 |
|  |  |  | Left Superior longitudinal fasciculus | Left | 4.06 | -44 | -44 | 0 |
|  |  |  | Supramarginal gyrus posterior | Left | 4.02 | -46 | -46 | 10 |
|  |  | 204 | Middle temporal gyrus posterior | Left | 3.66 | -68 | -6 | -16 |
|  |  |  | Middle temporal gyrus posterior | Left | 3.58 | -70 | -20 | -6 |
|  |  |  | Middle temporal gyrus posterior | Left | 3.37 | -70 | -26 | -14 |
|  |  |  | Superior temporal gyrus posterior | Left | 3.2 | -64 | -26 | -2 |
|  | Executive | 1584 | Lateral occipital cortex superior | Left | 5.06 | -34 | -80 | 18 |
|  |  |  | Forceps major | | 4.36 | -22 | -84 | 6 |
|  |  |  | Inferior temporal gyrus temocc | Left | 3.93 | -52 | -56 | -12 |
|  |  |  | Forceps major | | 3.83 | -28 | -70 | 16 |
|  |  | 200 | Left Putamen | Left | 4.22 | -32 | -12 | -8 |
|  |  |  | Left Amygdala | Left | 3.77 | -30 | 4 | -18 |
|  |  |  | Insular | Left | 3.56 | -38 | -6 | -14 |
|  | Speech quanta | 2135 | Central operculum cortex | Left | 4.77 | -58 | -6 | 12 |
|  |  |  | Precentral gyrus | Left | 4.61 | -64 | 0 | 28 |
|  |  |  | Precentral gyrus | Left | 4.43 | -60 | 0 | 18 |
|  |  |  | Postcentral gyrus | Left | 3.68 | -56 | -16 | 26 |
|  | Semantics | 8990 | Frontal orbital cortex | Left | 5.31 | -34 | 26 | -26 |
|  |  |  | Middle temporal gyrus temocc | Left | 4.91 | -46 | -50 | 10 |
|  |  |  | Temporal pole | Left | 4.81 | -46 | 24 | -20 |
|  |  |  | Lateral occipital cortex superior | Left | 4.54 | -32 | -74 | 14 |
| Extended (subgroup) | Phonology | 460 | Angular gyrus | Left | 4.19 | -48 | -58 | 56 |
|  |  |  | Supramarginal gyrus posterior | Left | 4.16 | -56 | -46 | 42 |
|  |  |  | Supramarginal gyrus posterior | Left | 4.11 | -60 | -54 | 44 |
|  |  |  | Postcentral gyrus | Left | 3.85 | -48 | -40 | 60 |
|  | Executive | 3289 | Lateral occipital cortex inferior | Left | 5.08 | -42 | -70 | 6 |
|  |  |  | Lateral occipital cortex superior | Left | 4.61 | -34 | -82 | 16 |
|  |  |  | Forceps major | | 4.21 | -24 | -84 | 6 |
|  |  |  | Left Inferior longitudinal fasciculus | Left | 4.1 | -32 | -74 | 2 |
|  |  | 191 | Brain Stem |  | 4.97 | -10 | -46 | -18 |
|  |  |  | Brain Stem |  | 4.6 | 2 | -48 | -20 |
|  |  |  | Brain Stem |  | 4.5 | 10 | -52 | -20 |
|  |  | 273 | Lateral occipital cortex inferior | Right | 4.71 | 36 | -80 | 2 |
|  |  |  | Occipital fusiform gyrus | Right | 4.36 | 32 | -72 | -14 |
|  |  |  | Lateral occipital cortex inferior | Right | 4.33 | 32 | -86 | -10 |
|  |  |  | Lateral occipital cortex inferior | Right | 3.63 | 38 | -80 | -16 |
|  |  | 830 | N/A |  | 3.95 | -16 | -78 | -34 |
|  |  |  | Brain Stem |  | 3.61 | -4 | -56 | -44 |
|  |  |  | N/A |  | 3.59 | -14 | -82 | -42 |
|  |  |  | N/A |  | 3.59 | -32 | -66 | -28 |
|  | Speech quanta | 581 | Precentral gyrus | Left | 3.89 | -64 | 2 | 24 |
|  |  |  | Central operculum cortex | Left | 3.75 | -60 | -6 | 12 |
|  |  |  | Precentral gyrus | Left | 3.66 | -56 | 2 | 30 |
|  |  |  | Central operculum cortex | Left | 3.17 | -60 | -20 | 16 |
|  | Semantics | 604 | Frontal orbital cortex | Left | 4.92 | -20 | 16 | -28 |
|  |  |  | Frontal orbital cortex | Left | 4.5 | -30 | 26 | -26 |
|  |  |  | Temporal pole | Left | 3.81 | -44 | 20 | -18 |
|  |  |  | Temporal pole | Left | 3.75 | -50 | 16 | -8 |
|  |  | 3199 | Left Inferior longitudinal fasciculus | Left | 4.54 | -44 | -44 | -8 |
|  |  |  | Supramarginal gyrus posterior | Left | 4.5 | -52 | -46 | 18 |
|  |  |  | Superior temporal gyrus posterior | Left | 4.47 | -60 | -34 | 4 |
|  |  |  | Superior temporal gyrus posterior | Left | 4.47 | -54 | -42 | 4 |
| Reduced (all cases) | Phonology | 250 | Supramarginal gyrus posterior | Left | 3.88 | -54 | -50 | 42 |
|  |  |  | Supramarginal gyrus posterior | Left | 3.67 | -62 | -54 | 40 |
|  |  | 227 | Left Superior longitudinal fasciculus | Left | 3.63 | -42 | -48 | 2 |
|  |  |  | Supramarginal gyrus posterior | Left | 3.52 | -46 | -46 | 10 |
|  |  |  | Left Inferior frontal occipital fasciculus | Left | 3.5 | -32 | -32 | 4 |
|  |  |  | Planum temporale | Left | 3.31 | -30 | -32 | 16 |
|  | Executive | 366 | Lateral occipital cortex superior | Left | 4.08 | -34 | -82 | 16 |
|  |  |  | Lateral occipital cortex inferior | Left | 3.62 | -28 | -84 | 6 |
|  |  |  | Left Inferior frontal occipital fasciculus | Left | 3.35 | -30 | -78 | 0 |
|  |  |  | Lateral occipital cortex inferior | Left | 3.34 | -40 | -76 | 8 |
|  | Speech quanta | 2208 | Central operculum cortex | Left | 4.72 | -58 | -6 | 12 |
|  |  |  | Precentral gyrus | Left | 4.59 | -64 | 0 | 28 |
|  |  |  | Precentral gyrus | Left | 4.39 | -60 | 0 | 18 |
|  |  |  | Precentral gyrus | Left | 3.73 | -38 | -2 | 44 |
|  | Semantics | 12106 | Left Superior longitudinal fasciculus | Left | 5.55 | -44 | -46 | 2 |
|  |  |  | Middle temporal gyrus temocc | Left | 5.51 | -46 | -50 | 10 |
|  |  |  | Frontal orbital cortex | Left | 5.04 | -34 | 26 | -26 |
|  |  |  | Temporal pole | Left | 4.96 | -40 | 22 | -22 |
| Reduced (subgroup) | Phonology | 494 | Postcentral gyrus | Left | 4.45 | -48 | -40 | 60 |
|  |  |  | Supramarginal gyrus posterior | Left | 4.4 | -56 | -46 | 42 |
|  |  |  | Angular gyrus | Left | 4.06 | -48 | -58 | 54 |
|  |  |  | Supramarginal gyrus posterior | Left | 3.5 | -50 | -50 | 58 |
|  | Executive | 169 | Precentral gyrus | Right | 4.3 | 24 | -20 | 60 |
|  |  |  | Right Corticospinal tract | Right | 3.81 | 16 | -26 | 58 |
|  |  | 450 | Lateral occipital cortex inferior | Left | 4.1 | -44 | -64 | -6 |
|  |  |  | Lateral occipital cortex inferior | Left | 3.91 | -42 | -70 | 6 |
|  |  |  | Left Inferior longitudinal fasciculus | Left | 3.69 | -36 | -66 | 2 |
|  |  |  | Left Inferior longitudinal fasciculus | Left | 3.65 | -32 | -74 | 2 |
|  | Speech quanta | 753 | Precentral gyrus | Left | 4.06 | -64 | 2 | 24 |
|  |  |  | Precentral gyrus | Left | 4 | -62 | 0 | 34 |
|  |  |  | Precentral gyrus | Left | 3.87 | -60 | -4 | 14 |
|  |  |  | Precentral gyrus | Left | 3.8 | -56 | 4 | 26 |
|  | Semantics | 9274 | Left Inferior longitudinal fasciculus | Left | 5.88 | -42 | -42 | -10 |
|  |  |  | Middle temporal gyrus temocc | Left | 5.04 | -46 | -50 | 10 |
|  |  |  | Middle temporal gyrus temocc | Left | 4.98 | -52 | -44 | -4 |
|  |  |  | Lateral occipital cortex superior | Left | 4.79 | -26 | -66 | 22 |
| CAT - all subtests (subgroup) | Phonological severity | 2476 | Supramarginal gyrus posterior | Left | 4.74 | -56 | -44 | 40 |
|  |  |  | Supramarginal gyrus posterior | Left | 4.5 | -60 | -54 | 44 |
|  |  |  | Supramarginal gyrus posterior | Left | 4.26 | -64 | -52 | 36 |
|  |  |  | Lateral occipital cortex superior | Left | 4.22 | -48 | -60 | 54 |
|  | Executive severity | 2690 | Lateral occipital cortex inferior | Left | 5.11 | -40 | -74 | 12 |
|  |  |  | Lateral occipital cortex superior | Left | 5.05 | -34 | -86 | 12 |
|  |  |  | Lateral occipital cortex inferior | Left | 4.99 | -28 | -86 | 6 |
|  |  |  | Lateral occipital cortex superior | Left | 4.82 | -40 | -66 | 18 |
|  |  | 149 | Precentral gyrus | Right | 4.97 | 22 | -18 | 56 |
|  |  |  | Precentral gyrus | Right | 3.68 | 28 | -26 | 52 |
|  |  |  | Right Corticospinal tract | Right | 3.66 | 22 | -26 | 46 |
|  |  | 158 | N/A |  | 4.88 | -22 | -42 | -48 |
|  |  |  | N/A |  | 3.89 | -34 | -46 | -46 |
|  |  | 172 | Lingual gyrus | Left | 4.78 | -20 | -56 | -4 |
|  |  |  | Left Hippocampus | Left | 4.36 | -32 | -36 | -8 |
|  |  |  | Left Cingulum | Left | 3.28 | -22 | -40 | -8 |
|  |  | 291 | Brain Stem |  | 4.45 | 0 | -32 | -24 |
|  |  |  | Brain Stem |  | 3.95 | 2 | -46 | -20 |
|  |  |  | Brain Stem |  | 3.84 | -12 | -44 | -18 |
|  |  |  | N/A |  | 3.45 | -6 | -50 | -14 |
| CAT - partial subtests (subgroup) | Phonological severity | 2123 | Supramarginal gyrus posterior | | 4.77 | -56 | -44 | 40 |
|  |  |  | Supramarginal gyrus posterior | Left | 4.49 | -60 | -54 | 44 |
|  |  |  | Supramarginal gyrus posterior | Left | 4.37 | -62 | -44 | 46 |
|  |  |  | Supramarginal gyrus posterior | Left | 4.33 | -64 | -52 | 36 |
|  | Executive severity | 6023 | Lateral occipital cortex superior | Left | 5.61 | -34 | -88 | 12 |
|  |  |  | Lateral occipital cortex inferior | Left | 5.52 | -40 | -76 | 12 |
|  |  |  | Lateral occipital cortex superior | Left | 5.08 | -40 | -66 | 18 |
|  |  |  | Lateral occipital cortex superior | Left | 4.82 | -38 | -76 | 20 |
|  |  | 263 | Brain Stem | Left | 4.65 | 4 | -34 | -24 |
|  |  |  | Brain Stem |  | 4.13 | -12 | -44 | -18 |
|  |  |  | Brain Stem |  | 3.96 | 2 | -46 | -18 |
|  |  |  | N/A |  | 3.68 | -6 | -50 | -14 |

Section 5.

We repeated the principal component analysis on the CAT using all the subtests or subtests that closely resemble those included in the extended battery. The results showed a two-factor solution for both variants and were highly similar. The Table shows the factor loadings for the two solutions. The two components from each solution were highly correlated: component 1 at r = 0.98 and component 2 at r = 0.88. The similarity between the two solutions was further confirmed in the VBCM analysis, where the neural clusters were almost identical with or without lesion volume correction (see Figure below). A direct comparison between the two models (using p to z conversion) did not reveal any differences.

Table. Factor loading table for two PCA solutions of the CAT. In the left columns, all tests were included and in the right columns we only included tests that overlapped with the extended battery (“-“ represents tests which were omitted in the right column).

|  | All-test | | Reduced-tests | |
| --- | --- | --- | --- | --- |
| Variance explained | 39.48% | 23.60% | 45.47% | 22.69% |
| Word Repetition | **0.917** | 0.139 | **0.901** | 0.229 |
| Object naming | **0.881** | 0.254 | **0.886** | 0.271 |
| Word reading | **0.875** | 0.261 | **0.899** | 0.233 |
| Action naming | **0.848** | 0.285 | **0.830** | 0.349 |
| Complex word reading | **0.833** | 0.312 | **0.837** | 0.317 |
| Sentence repetition | **0.813** | 0.231 | **0.772** | 0.316 |
| Complex word repetition | **0.799** | 0.262 | **0.763** | 0.349 |
| Digit span | **0.776** | 0.205 | **0.741** | 0.269 |
| Function word reading | **0.740** | 0.130 | **0.780** | 0.059 |
| Nonword reading | **0.736** | 0.248 | **0.776** | 0.160 |
| Nonword repetition | **0.731** | 0.111 | **0.665** | 0.273 |
| Picture description | **0.730** | 0.415 | **0.698** | 0.457 |
| Fluency Animals | **0.695** | 0.362 | **0.670** | 0.414 |
| Writing to Dictation | **0.686** | **0.519** | **-** | - |
| Fluency S | **0.625** | 0.331 | **0.600** | 0.378 |
| Spoken sentences comprehension | **0.608** | **0.528** | **0.564** | **0.615** |
| Written picture description | **0.556** | 0.460 | - | - |
| Arithmetic | 0.144 | **0.780** | **-** | - |
| Written word comprehension | 0.405 | **0.776** | 0.413 | **0.727** |
| Gesture object use | 0.183 | **0.752** | **-** | - |
| Writing picture names | 0.354 | **0.742** | **-** | - |
| Written sentences comprehension | **0.530** | **0.703** | **0.535** | **0.645** |
| Semantic memory | 0.236 | **0.666** | 0.210 | **0.682** |
| Spoken word comprehension | 0.397 | **0.662** | 0.364 | **0.713** |
| Copy | 0.390 | **0.597** | **-** | - |
| Recognition memory | -0.001 | **0.591** | -0.102 | **0.775** |
| Spoken paragraph comprehension | 0.429 | **0.547** | 0.340 | **0.739** |
| Line bisection | 0.044 | 0.376 | - | - |


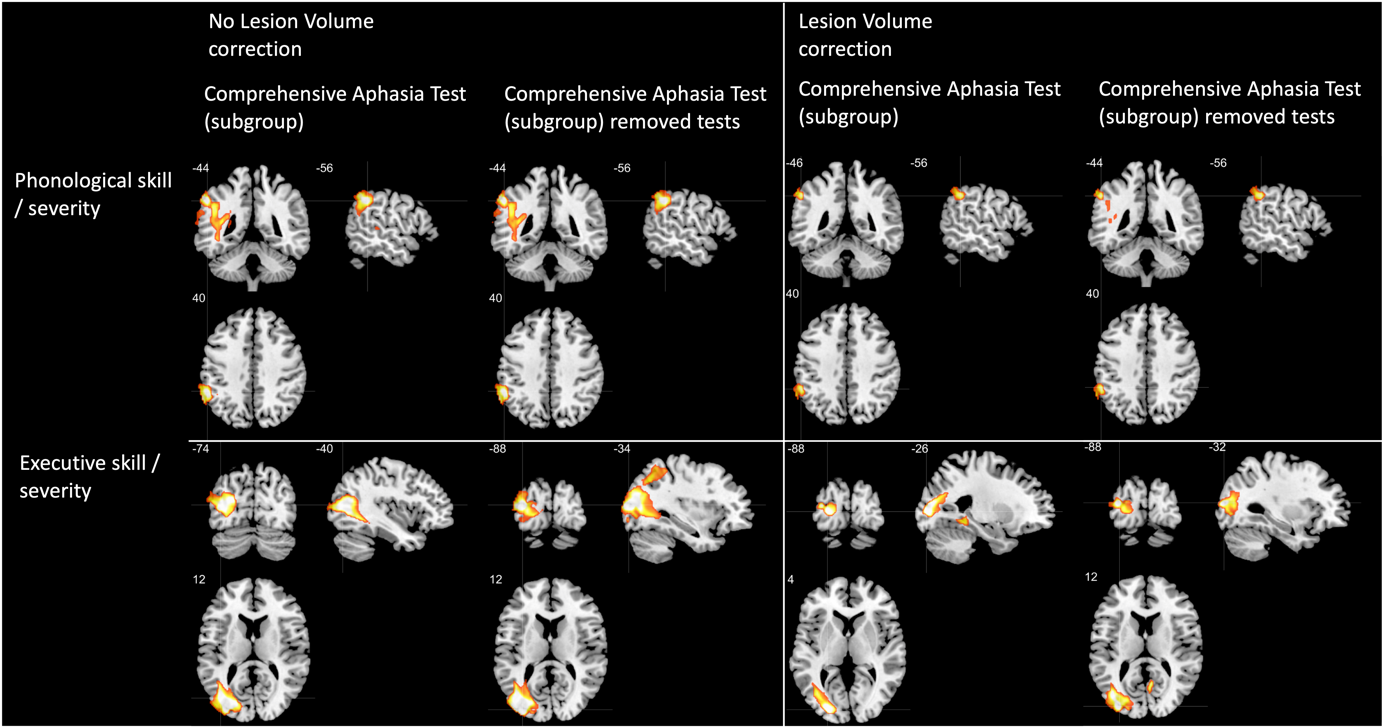


Figure. VBCM results for principal components extracted from the CAT for two different iterations. Firstly, components were extracted for all neuropsychological subtests (except the disability questionnaire; subtitled Comprehensive Aphasia Test (subgroup)). Second, all subtests domains that were not included in the extended battery (i.e., arithmetic, line bisection, object gesture use, and all writing/copy tasks) were removed and principal component extracted. The results indicate virtually no difference between the two component solutions (confirmed using direct contrasts, not shown). The results are thresholded using p < .001 voxelwise with family wise error cluster correction p < .05. The models with no lesion volume correction (left sided columns) have the following covariates: age, years in education, months post onset, and intracranial volume; while the right sided columns have the same plus lesion volume.
